# AI Methods in Sensor Calibration

**DOI:** 10.3390/s26092805

**Published:** 2026-04-30

**Authors:** Fei Kou, Yu-Qing Liu, Chen-Xi Li, Hong-Bo Qin, Yan Liu

**Affiliations:** School of Mechano-Electronic Engineering, Xidian University, Xi’an 710071, China; 24041212705@stu.xidian.edu.cn (F.K.); 24041212466@stu.xidian.edu.cn (Y.-Q.L.); 25041212558@stu.xidian.edu.cn (C.-X.L.)

**Keywords:** sensor calibration, artificial intelligence, transfer function, compensation

## Abstract

Artificial intelligence (AI)-based methods are rapidly advancing the development of sensor technology, bringing about significant advancements for sensors in structural design/optimization, fabrication, calibration and application. The recent involvement of AI models has provided a new paradigm for the calibration of sensors and greatly improved the accuracy and stability of obtained sensing characteristics. In this paper, we present an overview of the advances of AI methods in sensor calibration in recent years. The superiority of leveraging AI models in getting the transfer function, compensating for ambient interferences/drifts, and promoting large-scale, low-cost sensors is reviewed and discussed to illustrate the pioneering transformations in this domain. Relevant enhancing tools for data preprocessing, training optimization and data augmentation are also mentioned. The significant achievements in various sensing systems have demonstrated that AI methods can be a powerful solution to the critical issues in calibrating sensors. However, there are still several critical challenges persisting alongside these remarkable achievements, and long-term commitment remains essential for future investigations.

## 1. Introduction

In the current era, sensors have played a crucial role across diverse domains, including industry, agriculture, biomedicine, consumer electronics, air/water pollution monitoring, and smart home applications [1,2,3,4,5,6,7,8]. As an information provider, sensors should accurately capture the target measurands and reliably convert them into available signals for the subsequent processing and decision-making units [9]. This task requires an accurate transfer function for converting measurands into usable signals, and the function should remain invariant to different interferences in their operational cycles. Though the subsequent refining stages, such as signal amplification, filtering, and impedance matching, can enhance the quality, stability, and interoperability, they still work on the inherent input–output transfer function of sensors. Also, the extraneous signals caused by environmental interferences cannot be thoroughly eliminated. Therefore, calibration is an indispensable procedure for every sensor to establish a well-defined, robust transfer function prior to market deployment [10].

According to the official definition of calibration in JCGM 200: 2012 International vocabulary of metrology (VIM), the required first step is to establish a relation between the quantity values provided by measurement standards and corresponding indications, and the second step will use this information to establish a relation for obtaining a measurement result from an indication [11]. In the calibration, the standard stimulators with different magnitudes are loaded to the to-be-calibrated device with a certain interval, and the accompanying output signals (e.g., output voltage, capacitance, resistance) are recorded [12]. In order to map the input measurands and output signals with a linear pattern, the transfer functions for many sensors are designed to be linear [13]. However, there are still a substantial number of sensors with complex transfer functions, and their characteristic complexity is continually heightened by expanding application domains. Then, polynomial functions with more parameters are fitted to pursue the practical transfer functions. At this stage, the approximate fitting methods often rely on the simplified theoretical models or preset empirical models and the to-be-solved parameters are determined with the input measurands and corresponding output data [14]. The accuracy of these models and coverage capacity of captured data become the critical factors constraining the calibration precision [15]. For example, Zhang et al. compared the accuracy of fourth-order and second-order polynomials in characterizing the transfer function of a DC current sensor. Although the comparison was conducted under a fixed experimental ambient temperature, the average relative errors for current values (excluding 0.5 mA) still differed by 61.5% between the two models, while the discrepancy in relative error at 0.5 mA even exceeded 100%. Furthermore, experiments across a wide temperature range were not performed, precluding effective evaluation of the two transfer function models. Evidently, most of the preset models and their data cannot be in a sufficiently ideal status and the inherent deviation will cause errors for the sensors. Therefore, a more powerful tool is urgently needed to address this challenge.

Another core task for sensor calibration is compensating the influence of ambient interferences on the sensor performance, ensuring a robust transfer function across different operation scenarios [16]. In the operation surroundings, various factors, such as temperature, humidity, and the coupling effect from other measurands, have been proven to have an impact on the transfer function, making the established calibration relationship distorted [17,18,19]. Additional calibration efforts are required to address these interferences, in which corresponding disturbances of varying magnitudes are applied to explore the correlation between the interfering factors and sensor characteristics. Then, the effects can be compensated with subsequent hardware or software measures. However, the effectiveness of such compensation is also critically constrained by the accuracy of proposed influence patterns, and many approaches are only valid within a limited range. When facing the scenarios with more critical surroundings and requirements, the conventional approaches can only provide an approximate solution and further efforts are needed to improve the compensation for sensor calibration.

Furthermore, with the wide application of the Internet of Things (IoT) and consumer electronics, a large number of low-cost sensors (LCSs) have been deployed in air quality monitoring, water pollution assessment, agriculture, and wearable healthcare [4,20,21]. These devices often struggle to receive sufficient resources for high-precision transfer functions. Moreover, their characteristics are often more susceptible to environmental disturbances and prolonged usage can induce additional drift for these LCSs. Consequently, advanced calibration techniques for large-scale LCSs are also a promising direction, especially when powerful LCSs with extended operational lifespans and favorable accuracy are becoming necessary in the approaching intelligence era.

**Figure 1:**
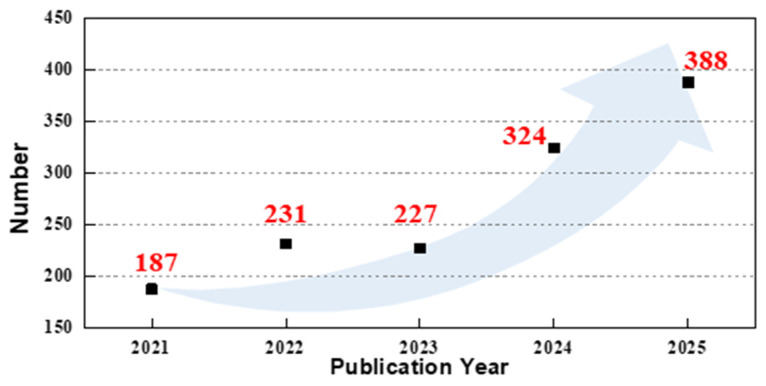
The publication year and paper number obtained from Web of Science Core Collection using “sensor calibration” and “artificial intelligence” as topic words.

In recent years, we have witnessed many ground-breaking achievements brought by AI across artistic creation, scientific research, and even our daily life. The exceptional regression and classification capability of machine learning methods and artificial neural networks have addressed numerous challenges in pattern recognition, trend prediction, and characteristic classification, demonstrating themselves as a powerful tool in many different domains [22,23,24,25]. AI methods also have posed tremendous applications in sensor-related scenarios [26]. Great advances have been revealed in the whole process of sensor research. For instance, the responses of sensing structure can be predicted in the design stage, defects can be inspected in the fabrication stage, and measuring features can be captured in the calibration stage [27,28,29,30,31]. Among these applications, sensor calibration is a typical process of regressing the relationship and identifying the influence pattern, where AI technology has demonstrated its significant advantage and emerged as a more powerful player. This is evident from the notable rise in publications containing the topic words “sensor calibration” and “artificial intelligence” in the Web of Science Core Collection over the past five years (2021–2025), reflecting a growing convergence of AI innovations with sensor calibration applications (Figure 1).

**Figure 2:**
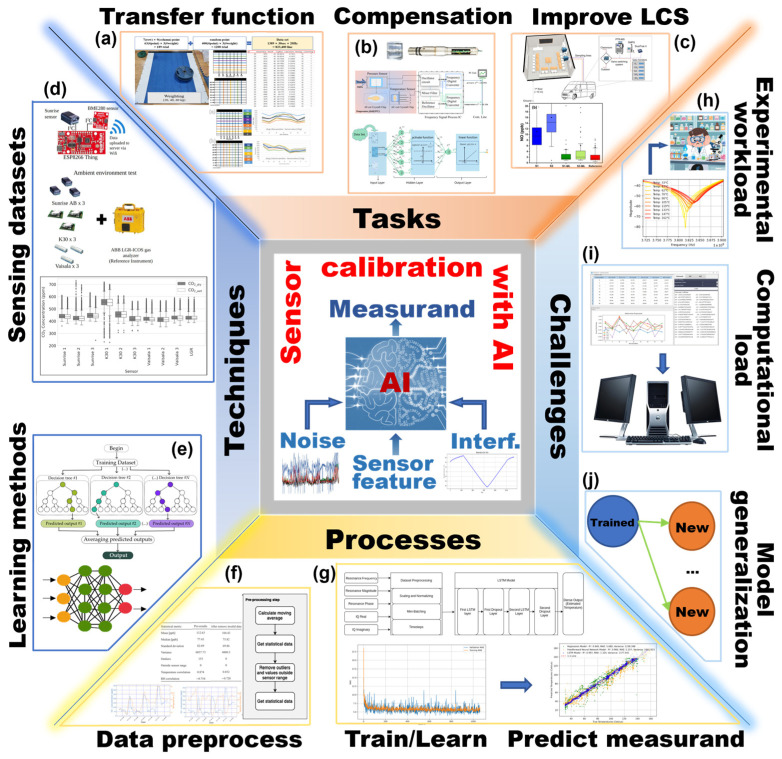
Summary for the AI methods in sensor calibration: (**a**) get the transfer function for the ground force sensor; (**b**) compensation of temperature for a quartz resonant pressure sensor; (**c**) calibrating the LCS for Indoor Air Quality; (**d**) the establishment of dataset for the CO_2_ NDIR Sensor; (**e**) the ML-based and DL-based learning models for sensor calibration; (**f**) data preprocessing before AI model training; (**g**) model training and measurand prediction (**h**) high workloads in acquiring experimental results for an antenna-based temperature sensor; (**i**) the computational load in AI model training and (**j**) the generalization of trained calibration model. All figures are reused under the Creative Commons CC BY license of [39,40,41,42,43,44,45]. Interf. = Interference.

With the emerging applications of AI methods in sensors, several excellent reviews have been published. However, most of them mainly shed light on the profound impact of AI across the whole development process, covering photoelectric sensors [32], gas sensors [33,34], microwave sensors [35,36], inertial sensors [37], and biosensors [38]. However, few of them pay sufficient attention to the AI methods in sensor calibration, and a systematic and in-depth review about the recent achievements, available methods, and possible challenges has not been effectively presented. As illustrated in Figure 2, this review will focus on the remarkable progress of AI methods in sensor calibration within the past five years (2021–2025), showing their capacities in constructing high-precision transfer functions, compensating influences of ambient interferences, and promoting the performance of LCSs. Meanwhile, the critical challenges in current research are also discussed. It should be noted that the calibration mentioned in this review only involves the process of obtaining the original information of measurands from sensor outputs, and the AI models referred to are still data processing strategies for sensor calibration. Although the physics of measurement constitute the most critical factor for calibration, the underlying principles, methodologies, and instrumentation are beyond the scope of this review. Also, further utilization of obtained information is not included, which can be found in the literature concerning intelligent sensing technology.

This review begins with an overview of sensor calibration in Section 2. Then, the application of AI methods in different calibration scenarios is systematically presented in Section 3, and some cutting-edge explorations for enhancing the performance of AI models are given, including data preprocessing, data augmentation, and training optimization. A discussion about the challenges is conducted in Section 5, and a brief conclusion for this paper is provided.

## 2. Overview of Sensor Calibration

### 2.1. Problem Statement

Calibration is a necessary step for sensors before they are put into the market. Generally, the characteristics of measurands are indirectly revealed by the outputs of used sensors. To obtain measurand information, a calibrated transfer function between the measurand and sensor signals is required, which can provide a practical model to convert sensor signals into meaningful measurand values. Simultaneously, the interferences in sensor surroundings (e.g., temperature and humidity) and performance drift after prolonged usage should also be fully considered. With necessary compensating measures, an accurate, reliable and stable transfer function is then obtained for the sensors. The calibration problem can be expressed as the following mathematical models, letting *x*(*t*) be the measurand profile, *y*(*t*) the sensor output profile, and *f*(.) the transfer function with undetermined adjustment coefficients vector *P* = [*P*_1_, *P*_2_, …, *P_n_*] that relates *x*(*t*) and *y*(*t*). Then, *y*(*t*) can be described as

(1) $$y\left(t\right)=f(P,x(t)).$$

In the calibration, the measurand profile is often provided by the standard/high-precision stimulation sources or high-grade sensors, and the output profile *y*(*t*) is captured by proper equipment. After proposing a suitable model for the transfer function *f*(.) and calculating *P*, the mapping model in Equation (1) is established. Then, the calibrated measurand profile *x*(*t*) is obtained by inverting the function:

(2) $$x\left(t\right)=f^{-1}(P,y(t)).$$

However, in practical scenarios, the undetermined *P* and even the form of *f*(.) can be affected by ambient interferences, and additional measurement errors are inevitably introduced in the whole signal transmission process. If we let *e*(*t*) be the measurement uncertainty related to random effects in the calibration, and considering the most common environmental factors of temperature (*T*) and humidity (*H*), Equation (1) can be rewritten as follows:

(3) $$y\left(t\right)=f\left(P\left(T,H\right),x\left(t\right)\right)+e(t).$$

Obviously, extra efforts should be devoted to investigating the features of *P*(*T*, *H*) and *e*(*t*), which can significantly increase the difficulty of obtaining *x*(*t*) from *y*(*t*). The accountment of additional factors (e.g., vibration, pressure, and electromagnetic field) will further enlarge the required data volume and dramatically increase the calibration experimental workload. Meanwhile, the construction of Equation (3) is no longer as straightforward as previously described due to the multiple involved factors.

Furthermore, the favorable performance of the aforementioned calibration is built upon the premise that the proposed *f*(·) is sufficiently accurate, which is also a hard task for many sensors. The pursued linearity at the design stage is often valid within limited ranges, and a much more complex form for the high-performance sensors is expected. The conformity between the preset *f*(·) and the actual working model of the to-be-calibrated sensor becomes the critical issue. However, the conventional calibration methods often use the simple polynomial functions as their *f*(·), and fit the obtained *x*(*t*) and *y*(*t*) data pairs to get the parameter P. This procedure is inevitably affected by the accuracy of the utilized approximate functional model and the coverage of data pairs over sensor characteristics. The favorable results may only be achieved after multiple iterative trials, especially when considering the introduction of environmental interferences and intrinsic errors. Therefore, more effective methods are still needed to improve the calibration accuracy and reduce workload.

Another critical issue for sensor calibration is handling the large volume of sensors with low cost, high speed, and acceptable accuracy. Currently, IoT and consumer electronics have deployed numerous low-cost sensing devices for better intelligence. These sensors also require proper calibration to fulfill their intended functions with fair measuring performances. However, the conventional point-by-point calibration for every participant is not practical due to the accompanying high resource consumption. Exploring suitable methods to address this issue is also a bottleneck on the path of advanced sensor calibration.

**Figure 3:**
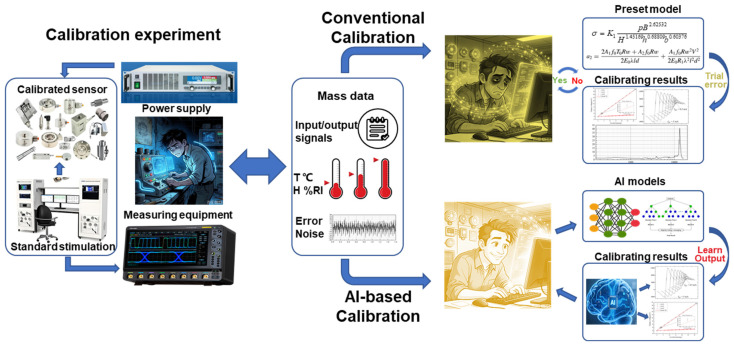
Overview for calibrating sensors with conventional and AI methods.

In summary, sensor calibration is a process of discovering and expressing the sensing characteristics with accurate transfer function, and minimizing the impacts from ambient interferences and possible errors. Thereby, the calibrated devices can exhibit a high precision and reliable sensing feature throughout their operational lifecycle. However, the conventional trial-and-error approach greatly depends on the iterations of preset models and extensive experimental verifications, and is a highly resource-intensive task for the production of high-performance sensors (as shown in the upper part of Figure 3).

### 2.2. Brief Introduction for AI Methods in Sensor Calibration

The introduction of AI methods in recent years provides a practical pathway for accelerating the calibration process and improving the obtained accuracy. Distinct from the abovementioned conventional approaches, AI methods possess superior capability in learning and extracting high-level features from large-scale data. They can effectively acquire the convoluted correlations between various parameters to lead to an unrestricted model that can replace the traditional *f*(·) with fixed expressions. Meanwhile, the influence patterns of environmental interferences on *P* can be recognized and then removed from the sensor output *y*(*t*), by which the effects of these interfering factors can be eliminated. The same paradigm is also viable for the measuring errors in the sensing system. With these subsequent compensating operations in calibration, the input–output relationship for sensors is restored to the simple form in Equations (1) and (2), though the *f*(·), *P*(*T*, *H*) and *e*(*t*) are not expressed in explicit analytical forms. The calibration tasks for large-scale LCSs may be treated by the strategy integrating the universal surrogate model with limited calibration data. As shown at the bottom of Figure 3, the AI methods have presented researchers with an important opportunity to adopt a more powerful and unconstrained style in processing data for sensor calibration. However, the AI method is still a data-processing operation that regresses the correlation relationship from obtained experimental calibration data, and the tasks discussed in this review remain those depicted on the right side of Figure 3. It remains powerless when confronting the issues beyond the scope of data processing. According to the definition of calibration in VIM, the measurement uncertainty is another critical parameter to evaluate the results. Regarding the uncertainties from principles and physical limitations, both conventional and AI methods are subject to identical constraints. Moreover, AI models are trained to minimize the deviations between the predicted measurand and the actual loaded measurand, but no explicit function can be extracted to evaluate the uncertainty during data processing. This issue is prevalent in current AI-based sensor calibration, and most research only pays close attention to the improvements of sensor accuracy induced by the utilization of AI methods. Therefore, this review focuses on the AI methods for getting the sensor transfer function and compensating environmental interferences, and the accompanying accuracy enhancements are the key evaluation metrics.

It should also be noted that AI is about creating machines or systems capable of performing tasks that typically require human intelligence, encompassing a wide range of domains and technologies. This paper primarily focuses on the machine learning (ML) models and artificial neural network (ANN)-based deep learning (DL) models in sensor calibration. However, this review will not be organized based on the employed ML or DL methods. Instead, we will structure the paper according to the scenarios encountered in sensor calibration, focusing on the addressable calibration issues. Various AI methods may be discussed in each calibration, and the details will be presented in the following sections.

## 3. AI Methods in Sensor Calibration

AI methods have played significant roles in various domains of sensor calibration, and remarkable achievements can be observed in getting the transfer function, compensating interferences, and treating large-scale LCSs. Moreover, the penetration of AI models is increasing with the improvement of measuring technology and sensor applications. This section will emphatically review the available AI methods in different calibration scenarios.

### 3.1. Get the Transfer Function

The primary target for the AI methods in sensor calibration is establishing the transfer function to reveal the relationship between the measurands and output signals. Along with the number of inputs perceived by a single sensing device, the operation complexity for AI methods also varies. The simplest case involves a single output signal for a single input quantity (referred to as single-to-single calibration), and the mapping relationship between the two can be accurately characterized by a well-trained AI model. It is the most frequent scenario for sensor calibrations. Another situation arises when the output signals are modulated by the profiles of multiple measurands (referred to as coupled calibration), which often requires more investment in establishing and training a more powerful AI model.

#### 3.1.1. Single-to-Single Calibration

Generally, the single-to-single calibration can be divided into two categories: (1) Devices are designed with a defined approximately linear feature, and their calibration aims at improving the characterization precision of proposed linear transfer function with a minimized measurement uncertainty. This can be found in many sensors for physical quantities, such as temperature, pressure, force and acceleration. (2) Devices feature highly nonlinear characteristics, and it is difficult to establish a high-precision transfer function for the conventional polynomial fitting methods. The calibration mainly focuses on discovering the implicit input–output relationships within the obtained experimental calibration data by utilizing the regression capability of AI models without imposing a preset constraint on the form of obtained function.

**Figure 4:**
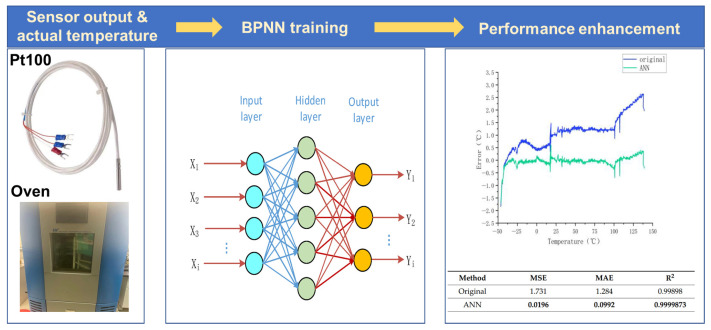
Workflow for calibrating a Pt 100 temperature sensor with BPNN. The figures are reused under the Creative Commons CC BY license of [46].

Herein, the temperature sensors are taken as an example to illustrate the implementation of AI methods in enhancing the precision of linear devices. The Standard Platinum Resistance Thermometer has a distinguished linearity from the triple point of water (0.01 °C) to the freezing point of Indium (146 °C) and is employed as the gold standard of thermometry. However, regarding the more common industrial Pt resistance temperature detector (RTD) in potentially wider temperature ranges, the linear models may fail to provide a superior characterization accuracy. In order to pursue a more favorable transfer function model for this situation, Liu et al. employed the Backpropagation Neural Network (BPNN) to learn the mapping relationship between loaded temperature and resistance values for a Pt100 RTD in the temperature range of −50~150 °C. The trained BPNN can directly predict the temperature based on the captured resistance, as shown in Figure 4 [46]. During the network training, the pairs of resistance value and corresponding loaded temperature serve as the dataset. With the highly linear transfer function obtained from BPNN, the squared correlation coefficient (*R*^2^) is promoted to 0.9999873, and the mean absolute error and mean square error are both decreased by two orders of magnitude. Though the improvement in sensor linearity and accuracy has been demonstrated, the practical value of using AI methods in these well-defined sensors is limited. The existing models for them are already sufficiently sophisticated, rendering AI participation merely a technical research alternative.

Compared with Pt RTD, the thermocouple often exhibits a poor linearity, and a stronger calibrating model can be more helpful. Anandanatarajan et al. addressed the inherent nonlinear characteristic of a K-type thermocouple with a deep feedforward neural network (DFNN) [47]. Three five-neuron hidden layers are utilized; the nonlinear voltage of the calibrated thermocouple serves as the sole input and the sole output gives the linearized temperature, forming a 1-5-5-5-1 architecture for the proposed DFNN. After determining the bias and weights using the multi-paradigm scripting language of MATLAB (MathWorks, Natick, MA, USA), the trained network reduces the nonlinear error of thermocouple from 2.03% to 0.002% within the temperature of −100 °C to 1372 °C. Meanwhile, a cold junction compensation is also conducted by using a linearized negative temperature coefficient (NTC) thermistor. A Deep Layer Recurrent Neural Network (DLR-NN) with an identical 1-5-5-5-1 architecture is adopted to deal with the inherent nonlinear characteristic of the NTC sensor. The resistance variation is converted into voltage by introducing a current excitation and fed into the network. Within the range of 0–120 °C, the nonlinear error of NTC is reduced from 84.63% to 0.13%, making it an excellent reference for cold junction compensation. With the help of the proposed linearization and compensation, the thermocouple can achieve a maximum absolute error of 0.34 °C within 0–300 °C when the ambient temperature varies from 0 °C to 40 °C, which greatly outperforms the existing National Institute of Standards and Technology (NIST) standards. The trained network can operate effectively on the Raspberry Pi 4 B+ platform, ensuring practical applications of the proposed method.

These works demonstrate that AI-involved calibration is a helpful strategy for further enhancing the precision of the sensors with approximately linear characteristics. Similar demonstrations can also be found in many other sensors, including the linearization of capacitive pressure sensor [48], the wireless patch antenna for temperature sensing [39], the unmanned air vehicle thermal sensor for agricultural application [49], the low-cost non-intrusive thermal flow meter [50], the sensor for thickness estimation of thin coatings on carbon fiber composites [51], the capacitive sensor for soil moisture measurement and type prediction [52], and the piezoelectric sensor for rainfall estimation [53].

As for the devices with high nonlinearity, the sensors for gas/air monitoring are representative. The highly nonlinear response characteristics make them frequently fail to meet the testing demands, especially under the heightened public attention to air quality [42,54]. Rapid and accurate determination of target polluting parameters from the sensor signals also constitutes a practical challenge for AI-based sensor calibration.

**Figure 5:**
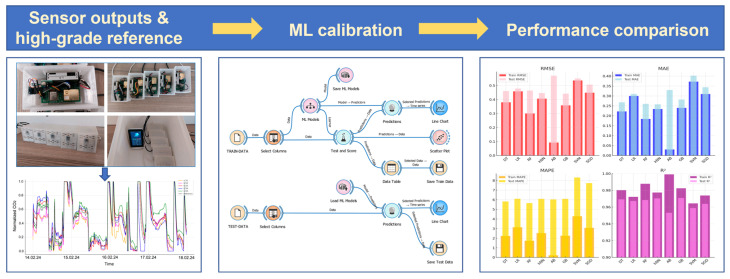
Workflow for the ML-based calibration of the PM2.5 and CO_2_ sensor. The figures are reused under the Creative Commons CC BY license of [58].

Herein, gas/air sensors are taken as the object to describe the AI methods for calibrating these highly nonlinear sensors [44,55,56,57]. Mehmet Taştan described the ML–Based Calibration of the PM2.5 and CO_2_ sensors in air quality detection [58], and eight different models are trained and assessed. The general workflow for sensor calibration is illustrated in Figure 5. The process begins with capturing and preprocessing the data of sensor measurements and reference values from high-grade equipment, which is then input into the ML models as the training/testing dataset. Performance metrics, such as *R*^2^, Root Mean Square Error (RMSE), Mean Absolute Error (MAE), and Mean Absolute Percentage Error (MAPE), are utilized to evaluate the capacity of each ML model in treating the calibration task. According to the metrics of multiple LCSs before and after ML-based calibration, the predictions of treated LCSs can more closely follow the trends of reference values. However, it should be noted that the performance of different ML models varies across the involved sensors. For the non-dispersive infrared LCS for CO_2_ (Model No. MH-Z19B, Zhengzhou Winsen Electronics Technology Co., Ltd., Zhengzhou, China), the Gradient Boosting achieves the best prediction. Then, the k-Nearest Neighbors produces the minimum error for the optical PM2.5 sensor (Model No. PMS7003, Plantower Technology Co., Ltd., Beijing, China). Similar phenomena are also mentioned by Dubey et al., who applied three different methods to calibrate three types of CO_2_ LCSs [43]. Obviously, a single calibration model cannot always fulfill the enhancing requirements from a certain type of sensors, and several attempts are needed before obtaining an appropriate algorithm.

**Figure 6:**
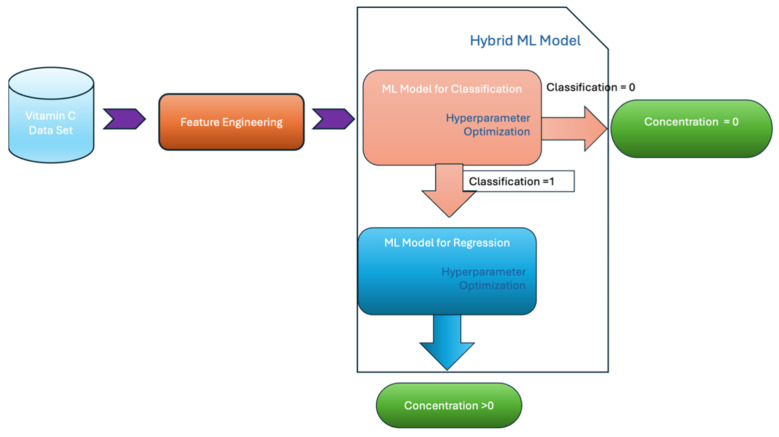
The two-stage hybrid procedure for calibrating a Vitamin C concentration senor. The figure is reused under the Creative Commons CC BY license of [59].

Recently, some improved approaches have also been explored to promote calibration performance. Decomposing the entire calibration into multiple substeps has been proved as a practical strategy. Different from common AI methods that directly map the transfer function using received datasets, the stepwise method partially accomplishes the tasks at each stage, and more effective information is passed to the subsequent steps for a further analysis. This approach can relieve the burden of each substep and simplify the whole calibration into several easy subitems. For instance, Dokuzparmak et al. employed a hybrid calibration model to achieve high-precision estimation of Vitamin C (VC) concentration with the data from their developed electrochemical sensor (Figure 6) [59]. A two-stage procedure is proposed with solution classification and concentration regression modules. The first stage uses the classification module to categorize tested samples based on whether VC is present, and the next regression stage focuses on the VC samples to predict the concentration. This arrangement can prevent the propagation of erroneous predictions, particularly in low-concentration or nondetectable samples, which are prone to signal noise and misclassification in traditional systems. A hybrid score considering classification accuracy, regression precision, and model stability is established to compare the model performances, and the CatBoost method is finally selected for both classification and regression. In the training, Bayesian optimization and grid search methods are employed for hyperparameter optimization to further ensure training efficiency. A two-step approach was also adopted by Allka et al. for the in-situ calibration and temporal pattern-based recalibration of the LCSs in IoT air quality monitoring platforms [60]. Firstly, the nonlinear ML models are used to process the instantaneous measurements to achieve an in situ sensor calibration. Subsequently, the daily temporal correlations in the data are considered and the recognized regular patterns of daily parameters (e.g., temperature or tropospheric ozone) in the database are captured and used to linearly map the in situ calibrated LCS data to the projected daily reference data to recalibrate the sensor. The test result indicates that the two-step recalibration can improve the estimates by up to 20–40%. Obviously, the extra temporal information can further improve the LCS calibration performance [61,62].

Though only the single-to-single calibration issue is discussed, the models referred to vary with the available data and involved sensors. It is impractical to set a universal guideline for the model selection of certain sensors. However, there is a relatively evident trend. If only instantaneous measurements are considered in the calibration, the simple regression algorithms—whether ML or basic ANN—can also achieve satisfactory accuracy; when the temporal correlating patterns are taken into account, the neural networks with more complex architectures (e.g., LSTM) are often required to discover the temporal correlations within long-sequence data. Furthermore, when the treated sensor possesses unusual output, the corresponding model also needs a special customization. For example, the mechanochromic sensor for strain or tensile monitoring needs a model that can treat the color distribution information [63], and the neural network for image processing can be helpful in calibrating the microfluidic paper-based uric acid sensor [64] and optical fiber-based force sensor [65,66].

#### 3.1.2. Coupled Calibration

Establishing the transfer function for the multi-parameter coupled sensor is a relatively complex task. The coupled calibration requires that the proposed methods can distinguish the impacts of various measurand components, and separately regress their transfer functions. Similar to the aforementioned single-to-single calibration, the coupled calibration also needs to address the precision enhancement of physical sensors by decoupling the multi-components and giving them an accurate transfer function. Moreover, the coupled calibration also faces the situation for biochemical sensor that different substances simultaneously affect the sensor response due to the limited selectivity. The following content will express the available AI methods in the two aspects of coupled calibration.

The multi-axis force sensor is a representative device for the first aspect. In the sensor, the multiple force components are often captured by the same elastic body, making the structural deformation not correspond to any single loaded force. The inevitable manufacturing errors further weaken the effect of the designed decoupling solution. Therefore, the component coupling is a critical factor for most multi-axis force sensors. The conventional method usually employs a linear model for decoupling, and the obtained precision is not always satisfactory. Therefore, AI methods have been gradually adopted for enhancing this parameter. To decouple the flexible six-axis force/torque sensor in massage therapy, Liu et al. utilized a tailored BPNN to address the nonlinear coupling features of the target sensor [67]. After determining the network architecture, a genetic algorithm is further employed to optimize the initial weights and thresholds of BPNN, which can improve the training efficiency and prevent it from falling into local optimization. After sufficient training, the obtained maximum Class I error of calibrated sensor is reduced from 2.9% F.S. to 1.27% F.S., and the maximum Class II error is reduced from 8.79% F.S. to 1.02% F.S., indicating a significant promotion in the decoupling performance. The errors can be further reduced to 0.751% FS and 0.603% FS by a deep neural network. When installed on a smart glove, the accompanying force/torque of massage therapy can be accurately acquired, which can provide a practical sensing device for replacing the professionals with intelligent equipment. Similarly, Chen et al. proposed an Informer network-based algorithm to address the coupling problem of the multi-axis wheel force sensor (MWFS) [68]. In the approach, a cooperating mode with multiple modules is adopted, in which the Decoupling Embedding layer focuses on the linear coupling issue of MWFS, and the Token Embedding layer and Informer encoder mainly extract the temporal coupling features in the sensor outputs. The highway network and fully connected layer conduct the decoupling compensation and output the accurate force components. With this framework, both the cross-axis coupling and time-varied coupling are effectively resolved. Experimental results demonstrate that the proposed method can reduce the maximum cross coupling error from the un-decoupled value of 16.18% F.S. to the decoupled value of 1.32% F.S. When comparing to the methods based on conventional extreme learning machine or BPNN algorithms, the proposed approach still exhibits a reduction of 41.12% for the cross-coupling error. AI-based decoupling calibration has also been applied to the sensor embedded in soft robots and bonnet polishing equipment [69,70].

The coupled calibration of biochemical sensors is often organized with two stages: identifying the targets and then predicting their concentrations [71]. In the practical situation, the sensor often needs to treat the mixtures of multiple substances with wide concentration ranges, and the exhibited responding characteristics often feature nonlinear profiles. Therefore, the calibration of this kind of sensor is much more complex than the abovementioned physical sensors. Recognizing the appeared gases needs highly selectively detecting capacity, which can be achieved by leveraging the distinct influence patterns of gases on a single sensor or sensing array. Fayos-Jordan et al. proposed a tiny-ML AI-based IoT e-nose system for hazardous odor detection and classification, which could discover the appearance of Ammonia, Bleach, Gasoline and Isopropyl Alcohol [72]. In order to be compatible with the limited resources of an on-site measuring scenario, a simple multilayer perceptron (MLP) network is proposed to identify the hazardous odors. Based on the data from a 64-channel odorimeter and ESP32 calculating platform, the model correctly classifies the vast majority of instances in each of the five classes, with only one misclassification error in misclassing bleach as isopropyl alcohol. In both the final validation and test phases, the model achieved an overall recognition accuracy of 0.9996 in the 2500 tested samples, and the recall/F1-scroe remains ≥0.999. The involvement of proposed model confers high-precision identification and hazardous gas alarming capabilities to the original systems that lack native classification functionality. However, this work mainly focuses on alarming the appearance of high-concentration odors by assessing the output of the odorimeter, and the real-time concentration measurement is not conducted due to the resource limitations of planned wireless, low-power, and low-cost applications.

**Figure 7:**
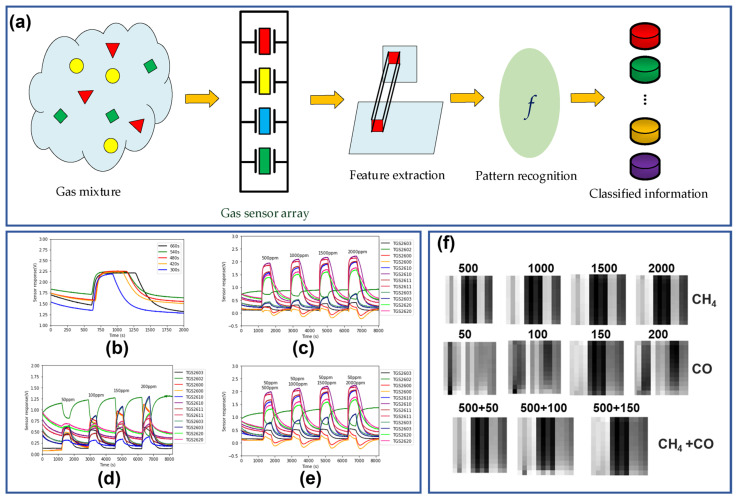
The calibration of gas sensor array with a LeNet-5 network: (**a**) the workflow; (**b**) the sensor response of CO at 50 ppm concentration at different injecting times; (**c**) response of 12 sensors to (**c**) CH_4_ at four concentrations, (**d**) CO at four concentrations, and (**e**) gas mixtures (50 ppm CO + 500~2000 ppm CH_4_); (**f**) obtained response patterns of the array with size 12 × 12 for different gases. The figures are reused under the Creative Commons CC BY license of [73].

A more complex calibration with the capacity of simultaneously completing species identification and concentration measurement was accomplished by Wei et al. [73], and the proposed frame diagram is shown in Figure 7a. An array with twelve metal oxide gas sensors is employed to identify and measure concentrations of two hazardous gases, CO and CH_4_, as well as their mixtures. The analyte injection time is settled at 480 s based on the responding curves with different times in Figure 7b, and the sensing responses vary with different gas parameters (Figure 7c–e) and are collected and then transformed into grayscale images with twelve stripes through normalization methods (Figure 7f). A LeNet-5 network is established to treat the obtained images for device calibration. It should be noted that the data from calibration experiments is not sufficient enough for network training, and the translation and cropping methods are utilized to expand them into a 1000-image dataset. The gas LeNet-5 can recognize the three categories of CO, CH_4_ and their mixtures omitting the concentration influences, and the final gas identification accuracy rate reached 98.67% with the unused data as a test set. Similarly, Aminaho et al. also employed ANN to get the gas component and concentration in CO_2_ capture and storage application [74]. Furthermore, with the help of convolutional neural network (CNN) models, Mei et al. established a transfer function for the VOCs sensor [75]. In this work, the temperature modulation technique provides a unique signature for each target gas, making it possible to treat different VOCs with a single sensor. The experimental data is first fed into the CNN for gas identification, and the corresponding concentration is predicted by the transfer learning model. The achieved identification relative uncertainty of ten VOCs is up to 98.38%, and the followed concentration predicting relative uncertainty is 88.65%, demonstrating the great potential of this approach in environmental and industrial monitoring [76].

Obviously, the participation of the AI model endows the coupling sensors with effective decoupling characteristics, and the improvements can be found in both physical sensors and gas mixture sensors. Commonly, the sensor targeting multiple parameters often has proposed the hardware-level decoupling at its design phase. Favorable decoupling or sensing selectivity can be found in the pressure–temperature dual-parameter sensor, capacitive six-axis force/torque sensor, piezotronics flexible tactile sensor and PVDF impact sensor for hypergravitational underwater explosion tests [77,78,79,80]. The hardware-level decoupling can eliminate the majority of coupling errors, but still remains imperfect due to material non-uniformity and manufacturing tolerances. Then, the software-level decoupling, such as the AI methods, will be a necessary complement. Currently, many coupled sensors employ both hardware-level and software-level decoupling simultaneously to achieve a better decoupling [81].

### 3.2. Compensation in Sensor Calibration

Ensuring the stability of the obtained transfer function under varying environments is also a critical issue for sensor calibration. Due to the inherent features of sensing principles and materials, nearly every sensor will exhibit certain types of drift when facing ambient temperature, humidity, or usage duration, and the determined transfer functions will also vary correspondingly. Meanwhile, the intrinsic errors in the whole sensing system can also affect the sensor outputs, bringing additional disturbances to the calibration. Therefore, the environmental interferences and errors must be fully considered, and the compensation for the induced deviations should be conducted. Concerning the common interferences during sensor calibration, this subsection will focus on the compensation for intrinsic error, temperature/humidity, and drift after prolonged usage.

#### 3.2.1. Compensation for Intrinsic Error

The systematic and stochastic errors of Inertial Measurement Units (IMUs) can severely impact the navigation performance, especially for the frequently used low-grade IMUs [82]. Therefore, the compensation of intrinsic error is essential for the calibration of inertial devices. The complex composition and patterns of IMU errors make it a well-suited target for AI methods. Herein, the AI methods for compensating intrinsic error will take IMUs as the example to illustrate their applications.

**Figure 8:**
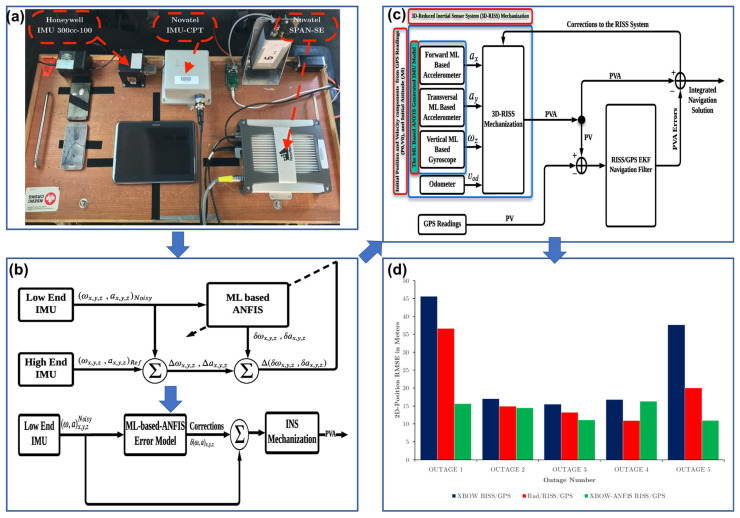
Compensation for intrinsic error in a low-grade IMU: (**a**) the experimental setup; (**b**) the training (**up**) and test (**down**) of proposed ML-based ANFIS; (**c**) the validation of proposed model in real navigations; (**d**) the obtained navigation error of different models. All figures are reused under the Creative Commons CC BY license of [84].

In practice, the output deviations between the low-grade (to-be-calibrated device) and high-grade (used as the ground truth) IMUs can be used as the training data for AI models to improve the performance of low-cost IMUs. Mahdi et al. employed a machine-learning-based Adaptive Neuro-Fuzzy Inference System (ML-ANFIS) to enhance the performance of low-grade IMUs, in which the ML-ANFIS is trained with the dataset of measurements from a low-cost IMU (XBOW IMU300CC, Crossbow, San Jose, CA, USA) and a high-end IMU (IMU-CPT, NovAtel Inc., Calgary, AB, Canada) (Figure 8a,b) [83,84]. In the tested navigation scenarios based on a proposed RISS/GPS system (Figure 8c), the leveraged low-cost IMU achieves a 54.23% and 51.14% reduction in RMSE azimuth when compared to the original XBOW RISS/GPS and conventional radar RISS/GPS implementations (Figure 8d). Significant enhancements in 2D/3D position accuracy and various velocity components are also attained. More recently, Sahu et al. proposed an Advanced Low-grade Inertial Measurement Unit framework (ALiMU) based on a fully connected feedforward neural network with attention-based mechanism [85]. The ALiMU can efficiently learn the nonlinear transformations required to map the low-grade inertial sensors to the high-grade IMUs. Compared to previously reported models based on LSTM, Regression Neural Network (RNN), Gated Recurrent Unit (GRU) or CNN, ALiMU can provide more favorable accuracy, robustness, reliability, and significant RMSE improvement to the calibrated device, demonstrating the superiority of the proposed AI model. Beyond high-grade IMUs, several other sources can also serve as the ground truth in IMU calibration. For instance, the robotic arms with a predetermined path can generate certain inertial components to stimulate the IMU calibration [86]. Additionally, the navigation data obtained from GPS can be a supplementary reference when the IMU is installed in an INS [87]. These achievements in enhancing the low-grade IMUs represent an emerging solution for using cost-effective alternatives to replace the high-end IMUs with desired high-precision inertial measurements.

#### 3.2.2. Compensation for Temperature/Humidity

Compensating for temperature and humidity variations is critical to ensuring the stable transfer function across different environments. For most sensors, humidity is a relatively stable factor, and the temperature often stays at the stage center. Meanwhile, the continuously operating sensors in air quality monitoring systems should pay attention to both factors due to their obvious variations throughout the day. However, the compensation for temperature and humidity is fundamentally similar. Therefore, we will focus more on the AI-based compensating methods than the specific environmental factor. Generally, the compensation can be divided into two categories based on the data format, namely the one with non-temporal data and with temporal data.

**Figure 9:**
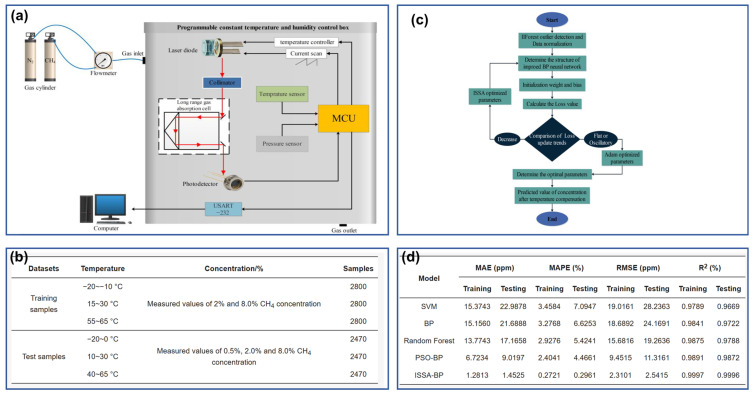
Temperature compensation for a laser methane sensor with improved BPNN: (**a**) the experimental setup; (**b**) the composition of obtained dataset; (**c**) the compensating workflow; (**d**) the compensation results of different models. All figures are reused under the Creative Commons CC BY license of [88].

In the compensation with non-temporal data, the interferences are also simultaneously loaded to the sensor at fixed intervals with the standard measurands, and the sensor responses under different interference amplitudes are captured. In this situation, all the values in the dataset are independent from each other and no temporal relationships exist, making the calibration process relatively straightforward. Yin et al. employed an enhanced BPNN-based model to address the temperature-induced fluctuations in the transfer function of a laser methane sensor, as shown in Figure 9 [88]. The sensor responses are collected at a 5 °C interval across the low (−20~0 °C), normal (10~30 °C), and high (40~65 °C) temperature ranges. A total of 15,810 data pairs under different temperatures and CH_4_ concentrations (0.5%, 2%, and 8%) are acquired for the model training and testing (Figure 9b). Experimental results demonstrate that the enhanced BPNN method can reduce the relative error of estimated CH_4_ concentration to 0.32% within the tested temperature range, exhibiting effective compensation for the temperature effects (Figure 9d). BPNN was also utilized by Huang et al. to conduct the temperature compensation for micromachined silicon resonant accelerometers, and the stability of the transfer function was significantly enhanced within the range of −40 °C to 60 °C [89]. In the full temperature range, the variation of the scale factor is improved by more than 70 times, and the variation of the bias is improved by around three orders of magnitude. The utilization of BPNN-like simple networks in interference compensation can also be found in the capacitive accelerometer [90], immune microwave sensor [91], multi-channel pressure scanner [92], mass flow sensor [93] and piezoelectric weight sensor [94].

When the involved temperature is a continuous time-series data, the models with limited processing capability are no longer suitable, and more powerful models are required to address the correlating relationships across the whole time series. Jiang et al. input both temperature and its change rate to the LSTM for processing the bias and scale factor error induced by temperature variations in a fiber optic gyroscope (FOG), and the FOG output was stabilized at 20°/s under thermal impact [95]. Furthermore, Ouyang et al. established an LSTM-SVM-DBN model based on the temperature and its change rate for the micromechanical gyroscope [96]. In the proposed method, DBN is employed to refine the compensation patterns predicted by LSTM and SVM, achieving a 95.57% reduction in bias instability across the temperature range of −40 °C to 100 °C. Moreover, the deep LSTM or bidirectional LSTM with multi-LSTM modules can jointly determine the final output through information transmission across different modules, and also have the potential of enhancing the temperature stability of calibrated sensors [97,98]. Additionally, some works separately compensated the static and dynamic effects of temperature in the time-series data. The static influence can be regarded as the abovementioned non-temporal situation, and its compensation also can be well-treated by the simple network models. Therefore, the compensation is then separated into two phases: simple networks for temperature static effects followed by the complex networks specifically targeting the dynamic features. Xue et al. implemented this concept by combining Variable Coefficient Regression (VCR) and LSTM models to respectively compensate the static and varied temperature patterns in a quartz resonant accelerometer [99]. Within the range of −40 °C to 80 °C, the temperature coefficients of scale factor and zero bias are greatly improved from 18,104 ppm/°C to 0.773 ppm/°C and 6713.5833 μg/°C to 3.5833 μg/°C, respectively.

In complex environments, simultaneously compensating multiple parameters may be required. The air quality sensors subjected to daily fluctuations in temperature and humidity represent typical application scenarios. Meanwhile, these sensors often operate in a periodic-data-transmission form, and the acquired data is also discrete. This means the data volume is relatively small, and various AI methods can be readily applied. For instance, Andrews et al. incorporated ambient temperature (T) and relative humidity (RH) parameters in the AI model training when calibrating the methane emission sensor [100]. The ppm errors are reduced to less than 1 ppm under the conditions of +2 °C temperature and +2.0% RH, ensuring the system reliability across diverse fields. In blind field tests, the calibrated sensors successfully detected 97% of all methane released and delivered a detection limit of 0.6 kg/h with a 90% probability of detection. Similarly, Apostolopoulos et al. also collected the T and RH parameters in the calibration of air quality monitoring systems for urban background site and classroom environments [42,101]. This approach significantly reduces the daily mean error across multiple sensors and ensures the reliability of measurement results. The consideration of temperature, humidity, and even atmospheric pressure can also be helpful when treating the LCSs for ambient air temperature and air particulate matter sensors [61,102,103,104,105,106].

It should be noted that the involved interferences in sensor calibration will vary across different application scenarios. In typical industrial environments, temperature is the most common factor with a large variation range and rapid change rate, and possesses a pronounced effect on sensor characteristics. But humidity often maintains a stable state with a small influence. Therefore, temperature compensation receives more attention from both the industrial and academic sectors. In air quality monitoring scenarios, sensors must operate continuously throughout the day in outdoor environments, where both temperature and humidity may undergo significant variations across different times and weather conditions. Therefore, the calibration procedure requires a simultaneous consideration of both parameters to ensure the result reliability.

#### 3.2.3. Compensation for the Drift After Prolonged Usage

The prolonged usage can change the transfer function of sensors and cause a drift in the characterizations, which is more critical for biological or chemical sensors [107]. If this drift is not well compensated and the transfer function is not updated in a timely manner, possible deviations will occur in the measuring operations. To address this kind of long-term drift, the AI methods have been utilized to detect and identify these deviations and eliminate their influences from sensor outputs. The conducted drift compensation enables the sensor to maintain satisfactory accuracy over extended operational periods.

**Figure 10:**
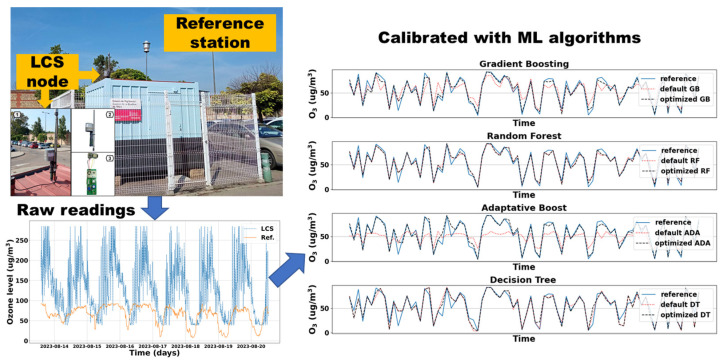
Calibrating the O_3_ sensor based on the historical data and ML mothos. All figures are reused under the Creative Commons CC BY license of [108].

Electronic nose (e-nose) is an advanced sensing system with selective chemical sensor arrays and appropriate recognition methods that can identify simple and complex odors. However, it often suffers from aging-induced drift, posing significant obstacles to its large-scale and widespread application. To address the precision maintenance requirements of the O_3_ sensor in an air quality module (ZPHS01B), Montalban-Faet et al. established two datasets consisting of 165- and 239-day test results to pursue the patterns of accumulated drift over these periods (Figure 10) [108]. Experimental results demonstrate that the raw continuously operated data from uncompensated LCS exhibits an evident offset in the measuring feature and produces enlarging deviation from the air quality parameters of the regulated reference station. The situation is so serious that the presented data has lost its value in presenting the true O_3_ concentration and can only show the daily change trends. Then, a ML-based compensation was conducted, aiming at substantially suppressing this deviation-induced offset. The determination coefficient between ML-compensated data and reference data is improved by over 250%, and the estimation error is reduced by approximately 90%, validating the excellent efficacy of drift compensation.

**Figure 11:**
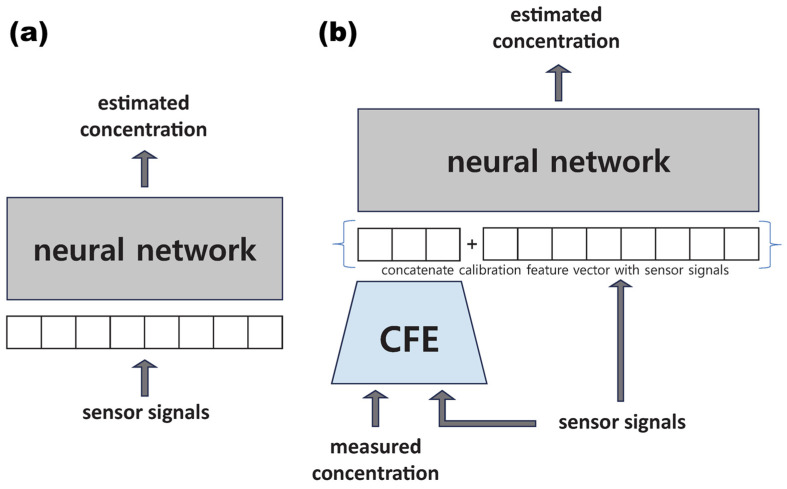
Comparison between the (**a**) conventional neural network-based method and (**b**) neural network with a CFE method. The figure is reused under the Creative Commons CC BY license of [109].

Meanwhile, the operational paradigm of extracting drift features prior to conducting compensation has emerged as a more prevalent approach. Kwon et al. did not directly feed the sensor output into the AI model in the compensation. Instead, they first employed a Masked Autoencoder Module to concatenate a calibration feature vector (CFV) from past sensor data and used this as an input to the neural network [109]. A comparison between the conventional neural network-based method and the neural network with a calibration feature encoder (CFE) method is shown in Figure 11. In the proposed model, a 128-dimensional masked autoencoder, namely CFE, is proposed to extract the CFV. With the assistance of obtained drift features from CFV, the employed network can fulfill the compensation task with a small amount of field test data, and accurate gas concentrations can be rapidly predicted. This scheme fully uses the drift features hidden within historical data, and avoids the extensive data collection and tiresome retraining in real-time calibration. Evaluation of a 3-year gas sensor array drift dataset demonstrates that the model with CFE can achieve substantial performance improvement for all six participating sensors. Compared to the conventional fine-tuning neural network, the obtained RMSE for concentration prediction is further reduced by an order of magnitude. Similarly, Liu et al. constructed a metalearning-based adaptive in-field calibration (MAIC) framework for IoT air quality monitoring systems [110]. Firstly, the metaknowledge is extracted from the historical data by developing task generation strategies and proposing task-oriented model training. Then, an adaptation method is presented to learn the in-field calibration data distribution without forgetting the metaknowledge, enabling continual learning to utilize the temporal dependencies between multiple conditions. The model can learn continuously instead of training from scratch and alleviate the overfitting problem when adapting with limited data, ensuring a favorable generalization ability. The results show that the MAIC outperforms many other conventional calibration algorithms in the real-world deployment data set, while requiring significantly fewer in-field reference data points.

Additionally, the test data can be pre-scanned to eliminate low-importance data points, which facilitates the acquisition of more precise information during feature extraction. Following this rationale, Guo et al. introduced the attention mechanism to screen the specific features of the gas data and remove the low-weight features before the feature extraction network [111]. Then, the combining of the segmented training method and targeted cyclic training model of CNNs further reduces the required experimental data for drift compensation. Validated with the largest existing gas drift dataset, the proposed method maintains the average gas detection accuracy beyond 80% in three years, and the long-term stability of gas detection is effectively improved.

The aforementioned works have proved that the principal task in drift compensation lies in extracting the drift patterns from sensor time-series outputs. The participation of long-term historical data is an important assistance for this work, and the AI methods capable of handling this data are further required to ensure compensation accuracy.

### 3.3. Calibration of Large-Scale LCSs

In the production and application of sensors, the rapid calibration for large-scale, low-cost sensors also requires dedicated effort. The calibration of individual high-performance devices has been achieved by the abovementioned studies, but these tailored methods may become powerless when scaling to the large-scale LCSs. Substantial calculation and experiment resources are invested, increasing the product cost. Obviously, this approach is not suitable for the suppliers or end-users who are seeking appropriate measures to achieve favorable calibration for large-scale LCSs without a large cost. Furthermore, the generalization capability of the AI model will become worse when it greatly focuses on a specific device, and cannot be directly reused in other scenarios [112]. There is still a pressing demand for AI calibrating methods specifically designed for large-scale LCSs, which can comprehensively balance performance improvement against cost.

**Figure 12:**
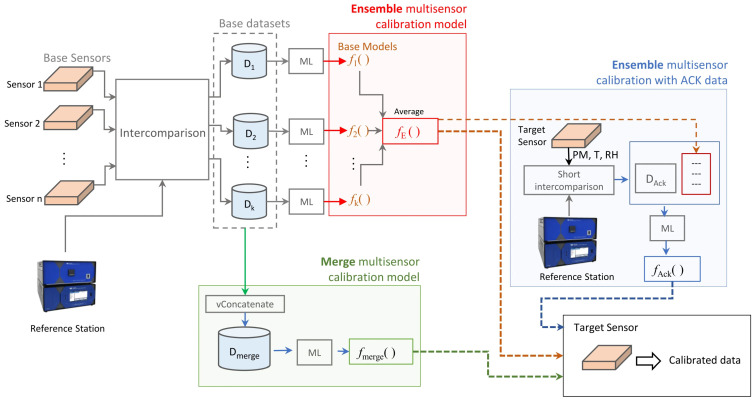
The transfer function model proposed for multi-sensor calibration modeling. The figure is reused under the Creative Commons CC BY license of [115].

Many researchers have adopted transfer learning approaches to address this challenge. The pre-learned knowledge is migrated to a new to-be-calibrated device with the help of a small amount of supplementary experimental data [113]. Adoui et al. established a distinctive construction methodology for the learning dataset to tackle the calibration of large-scale air sensors [114]. In the proposed dataset, the data for ML training is sourced from high-precision sensor systems, but the test data originates from the sensors under calibration. The trained ML model is then utilized to predict the measurands from LCS outputs. According to the validating results, the improved random forest method can achieve satisfactory prediction performance, with an *R*^2^ score of 0.72 and RMSE of 0.0028 ppm. This approach may possess potential for the LCSs with low-resource microprocessors. However, this method cannot well cover the full characteristics of the calibrated sensor, and the calibration accuracy is not very desirable. The more prevalent methodology is training a comprehensive model with a generic historical dataset to establish an initial transfer function, and the model is then refined with the additional features from limited experimental data. Villanueva et al. leveraged a sensor transfer function model derived from existing field data of several devices to build the calibration model for new sensors from the same manufacturer and thus facilitate their rapid incorporation into the air particulate matter monitoring network (Figure 12) [115]. In this work, the data from base sensors and the reference station are processed through ML methods and then averaged or merged to construct a generic calibration model for this sensor batch. The obtained models will be optimized by incorporating limited real testing data to achieve a more precise calibration result for the new sensor. Based on the results from IQAir PM10 sensors, the original calibration model from averaging or merging operations has shown acceptable prediction errors. After introducing the correction data from calibrated sensors, the RMSE and *R*^2^ can be further improved. The similar approach involving is also employed by Amagai et al. to calibrate the robotic distance sensor arrays [116].

This transfer learning here does not mean applying the same trained model to every new sensor, which would yield negative effects due to the possible gap between the source and target domains. Additional calibration data from the target sensor is also required to ensure the desirable accuracy. In practice, an accumulated historical dataset is leveraged to primarily train the AI model, and a small amount of experimental data from the target sensor is acquired to refine the model. The model based on historical data represents the general characteristics of a certain product, while the participation of newly acquired measurement data reveals the individual specifics. This two-stage transfer learning can reduce the required data volume for each calibration, and then alleviate the experimental burden and operating cost. Similar to other calibration methods, the compensation effect depends on the consistency between calibration conditions and application conditions. Therefore, the calibration condition must be established after investigating the detailed application environments and identifying the core influencing factors, which will be incorporated into calibration experiments. If significant changes occur in the application scenarios, the calibration procedures must be reformulated.

Beyond the aforementioned calibrations, AI methods have also facilitated proper calibrations for many specialized scenarios without mature measuring methods. The examples include bone healing stage monitoring and assessment based on RF devices [117], flavor blueprinting of tea infusions [118], continuous biomass measurement for plant growth [119], on-site detection and discrimination of mycotoxins in corn samples [120], and plant dehydration monitoring [121]. The measurands referred to are difficult to precisely acquire by traditional sensing approaches. The incorporated AI methods can play their powerful regression capability to construct a highly accurate transfer function for the involved device and measurand, making the hidden parameters clearly displayed.

## 4. Enhancing Tools for the AI Methods in Sensor Calibration

In the application of AI methods, several supplementary tools also have played important roles in enhancing training efficacy and calibration accuracy. Presently, the available enhancing tools mainly focus on preprocessing raw sensor data, optimizing the model training parameters and augmenting the dataset. The following parts will provide a detailed introduction for these three aspects.

### 4.1. Data Preprocessing

In sensor calibration, the available data is often derived from standard exciting equipment and the sensor outputs. This data can exhibit the characteristics of large volume, containing interferences, diverse components, and varied magnitudes. If they are directly input into AI models for training, the obtained results may be compromised due to the accompanying low efficiency, local overfitting and degraded prediction. Therefore, many researchers will specifically introduce a preprocessing procedure before the model training.

**Figure 13:**
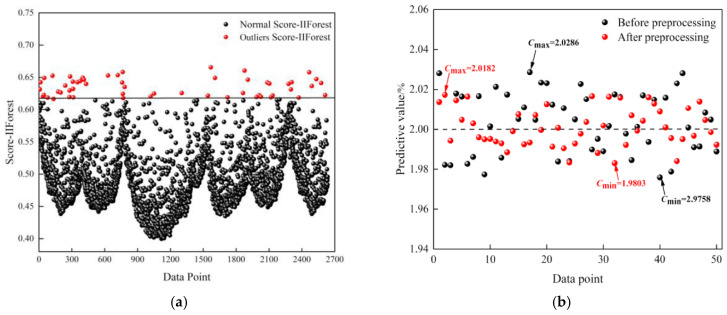
The improved iForest in data cleaning for 2% CH4 gas concentration data: (**a**) anomaly rating scores, (**b**) comparison of concentration prediction results concentration before and after data preprocessing. All figures are reused under the Creative Commons CC BY license of [88].

The most critical issue for data preprocessing is the detection and cleaning of abnormal data to avoid the influence of outliers in sensor data. The methods can be divided into traditional statistics-based approaches and AI-based methods. Few studies also use the simple neighboring averaging method to eliminate obvious outliers [45,122]. Generally, the conventional statistics-based methods such as the Inter-Quartile Range, have a low computational overhead and rapid processing speed [48,74]. However, they perform effectively only on the data of certain distributions, and exhibit poor performance with the high-dimensional data. More recently, machine learning approaches have frequently been adopted to address this issue, and clustering modules have also been introduced to further enhance detection efficacy. For instance, Yin et al. integrated the K-Means++ clustering algorithm into a traditional Isolation Forest (iForest) framework, mitigating the ineffective and low-precision segmentation caused by random value selection of the iForest algorithm [88]. Firstly, the basic iForest is employed to establish a set of decision trees, and the K-Means++ is utilized for automatically selecting the cluster number. The clustering result is then used as a branch of the isolated forest tree. This scheme allows for better differentiation between each cluster and provides more accurate anomaly scores, which become the criterion in the data cleaning (Figure 13a). The achieved temperature compensation for laser methane sensor with data cleaning demonstrates the significant improving effect of data preprocessing. As illustrated in Figure 13b, the predicted concentrations with preprocessed data (red points) are much closer to the actual values compared to those from raw data (black points).

Identifying and eliminating the systematic noise in the sensor signals is another essential task for data preprocessing [53]. For instance, Aldelemy et al. employed wavelet transforms to decompose noisy signals of the RF sensor into different frequency components, isolating and preserving the critical features while suppressing high-frequency noise. The smoothing filters were then proposed to effectively reduce noise impact [117]. Chen et al. utilized the smoothness priors approach (SPA) to remove the random noises and extract data trends for a dissolved gas sensor [123]. The measured gas parameters cannot change rapidly, and the derivatives of the data also exhibit limited variation rates. As a straightforward and effective detrending strategy, SPA can efficiently eliminate the high-frequency noise with a small window size. It is evident that the mentioned denoising measures primarily aim at the obvious anomalous noises, which are readily identifiable and weakly coupled with sensor outputs. This fundamentally differs from the error compensation in the above subsection. The corresponding elimination mainly works as an assistance to reduce the feature volume in model training.

In order to further facilitate the model training, the cleaned data may also need to experience the normalization and dimensionality reduction. Normalization mainly uses the maximum–minimum scaling method to yield a dataset with values bounded between 0 and 1. Additionally, when the dataset possesses the required distribution, the standard deviation-based scaling can also be applied [124]. For dimensionality reduction, principal component analysis (PCA) is a popular operation. For example, PCA is used to linearly transform rearranged |S_21_| data of microwave sensor before inputting it into the neural network, describing the data variation with fewer dimensions than the original representation [51].

### 4.2. Optimizing Algorithm for Model Training

**Figure 14:**
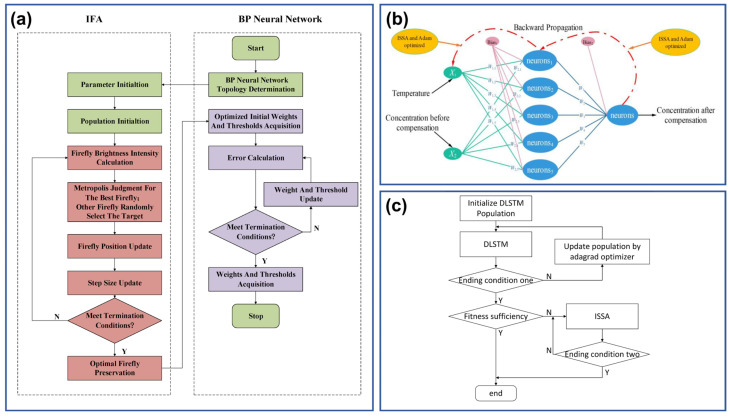
The AI models with optimizing algorithms for improved training: (**a**) the BPNN with IFA, (**b**) the BPNN with ISSA and (**c**) the DLSTM with ISSA. All figures are reused under the Creative Commons CC BY license of [88,89,97].

The model training involves the configuration and optimization of multiple network parameters, and the inappropriate initial parameters may lead to slow convergence and insufficient accuracy. Therefore, some optimization algorithms, such as the Genetic Algorithm (GA) and Particle Swarm Optimization (PSO), are often introduced to fine-tune the network initialization. The BPNN, often used for temperature compensation, is a kind of model that suffers from the low learning rate and susceptibility to falling into the local minimum if the initial weights and thresholds are improper. These iterative optimization algorithms can globally optimize the initial parameters before the formal training of the enhanced BPNN begins [94]. Taking GA-BP as an example, the constructed BPNN is not commenced directly with preset initial parameters. Instead, GA is first utilized to iteratively optimize the weights and thresholds based on the calculated fitness. Upon satisfying the termination conditions, the corresponding weights and thresholds are transferred to BPNN for formal training [125]. Many other optimization tools have proven their superiority in model initialization, and the reported algorithms include Improved Firefly Algorithm (IFA) (Figure 14a) [89], improved Sparrow Search Algorithm (ISSA) (Figure 14b) [88], Particle Swarm Optimization (PSO), and Global Search Artificial Bee Colony [126]. The involved Neural Networks have also spread to LSTM (Figure 14c) [97], Generalized Regression Neural Network (GRNN) [127], and many other AI models.

### 4.3. Data Augmentation

The training efficacy of AI models depends not merely on the used architecture and parameters, but critically necessitates high-quality datasets to provide the learning materials. However, the calibrating experiments often consume substantial resources in manpower, time, and consumables. This burden is further exacerbated in the calibration of biochemical sensors, due to the great investment in preparing multiple samples and waiting for stabilization and measurement in every calibration point. Coupled with the extensive data required by AI models, this poses a considerable challenge for research endeavors [27]. Therefore, researchers have been investigating alternative approaches to endow AI models with adequate input–output data.

Firstly, the physics-based modeling of sensors is a possible source for the calibration data [128]. When calibrating the cantilever-beam resonator for characterizing flow, temperature, pressure, and gas, Ghommem et al. utilized finite element simulation to produce the sensing responses (e.g., natural frequencies, quality factors, and static deflection) under different measurand excitation. The generated input–output pairs become a supplement for revealing the transfer relationship with AI models [129,130,131]. However, the finite element simulation cannot perfectly align with the actual experimental prototypes. Additional validation or refinement is also conducted based on real testing results to improve the conformance, forming a physics and data co-driven strategy for this task. The verified function can also work as the physics-based model. Britez et al. proposed an empirical function for the relationship between soil moisture and capacitance variation, and sufficient data is obtained from this physical model for training the AI model [132]. This method significantly improves soil moisture measurement accuracy with 84.83% of sensors showing improvement, offering a more agile and cost-effective implementation compared to traditional approaches. Another path for a physics and data co-driven strategy is proposing a theoretically fitting formula with the coefficients determined by limited prototype experiments [133]. The coefficients can be solved by the AI models to achieve accuracy and efficiency. This approach can reduce the volume of required measurement data while diminishing the prediction error by nearly an order of magnitude. All these investigations employ a data generation method to augment the experimental data for sensor calibration. However, the trained AI models with these generated data should undergo a refinement with some real calibration data to guarantee the precision [134].

**Figure 15:**
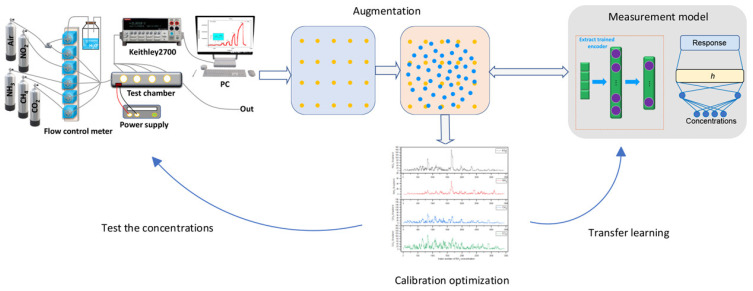
The workflow for the data augmentation based on AEN and VAE for calibrating GSA. The yellow points indicate the original data, and the blue ones for the augmented ones. The figure is reused with permission from [135].

Another approach to augmenting training data is learning sensor characteristics from historical data and generating synthetic data that mimics these learned patterns. For instance, Ma et al. leveraged the autoencoder network (AEN) to extract features from historical experimental data, and a variational autoencoder (VAE) is then utilized to generate additional data resembling the distribution of experimental data, as shown in Figure 15 [135]. In the calibration of the gas sensor array (GSA), only a small subset of test points across large-scale GSA deployments is captured to fine-tune the model based on generated data. In practical validation, the developed method effectively distinguished concentrations of four mixed gases. Similar methods have also been applied to calibrate the methane sensor [136,137], MEMS-based inertial sensor [138,139], and fiber optic sensor in pipeline monitoring [140].

Furthermore, directly augmenting the experimental data is also workable. There are two possible paradigms for this domain: introducing noise into existing data and generating additional data that aligns with experimental results. The former approach often superimposes random noise to the available experimental data. For example, Gaussian noise is added to the experimental results of VOCs in human breath, which can simulate the minor random fluctuations in sensor response and then address the potential model overfitting and the generalization to unseen scenarios [141]. Encoding technique is a popular approach for generating nonexistent data with existing experimental data, whose strategy is similar to the above historical data-based approach. The Variational Autoencoders [142], Convolutional Variational Autoencoders [143], and even Generative Adversarial Networks [144] have been demonstrated as effective augmentation methods. Essentially, the augmentation of existing experimental data serves merely as a reinforcement of the features already present within the available data to improve the robustness and generalization, but no new information is provided for the construction of the transfer function. The original data still needs to cover the full measuring range and critical characteristics.

## 5. Discussions and Challenges

This paper has presented a systematic review of the AI methods in sensor calibration, and several typical works are further summarized in Table 1. With the participation of AI models, many sensors with complex sensing characteristics and high interference sensitivity can achieve a high-precision, high-stability transfer function. However, not all AI-assisted calibrations yield practical value. For the well-defined sensors, the performance improvement from AI methods is often marginal. The illustration can be found in the utilization of BPNN in Pt RTD. The existing standardized characteristics already enable high accuracy through a simple linear regression, and the new R^2^ shows an improvement of merely 10^−5^—a negligible gain for practical applications. Conversely, for the sensors lacking accurate models, AI can significantly enhance their measurement performances. Significant practical value is realized when AI models address the high nonlinearity of gas sensors or NTC thermistors, as well as the complex interference patterns from the environment. Essentially, a more intractable transfer feature in the sensor can bring a higher value to the intervention of AI methods. Moreover, two additional issues should be further explained. Firstly, this review does not strictly differentiate the utilization of ML or DL methods, and the core focus is on the problems that AI models can solve. Herein, a simple discussion about the ML and DL models is conducted. Generally, the ML model is often used to treat the problems with small data volume and simple correlation due to its limited regression and classification capability, and the required resource is also limited, making it suitable for the simple single-to-single calibration or IoT nodes. Meanwhile, the DL models, especially those with complex architectures, can address the sophisticated correlations within a large volume of data, and the model training often requires sufficient resources. Therefore, it is very active in the calibration with temporal features or high-grade precision requirements [145,146]. There are no fixed criteria for model selection, but careful consideration should be given to balancing task complexity, expected accuracy, and available sources. Furthermore, establishing a universal rule for selecting specific methods from different ML/DL models is also challenging. A model may achieve optimal results in one sensor, but exhibit degraded accuracy when applied to another similar device. The performance of a certain model can be limited by several factors, and pursuing the so-called best model is more challenging. A general guideline can be provided. When processing the non-sequential data without temporal patterns, the ML and simple ANN approaches are efficient enough to obtain satisfactory calibrations. Meanwhile, when processing the large-scale time-series data, the models with enhanced regression capability, such as LSTM or CNN, often produce a superior performance.

Although many milestones have been achieved, AI methods in sensor calibration still require persistent devotion to address the following possible challenges:

(1) Acquisition of labeled data requires a substantial resource consumption. The AI-based calibrations greatly rely on the support from labeled data, which requires high-precision standard loads to stimulate the sensor and sophisticated equipment to capture the sensor outputs. The produced information is then used as the labeled input–output pairs for model training. All these devotions become a mandatory investment in the sensor calibration. Furthermore, a significant amount of manpower, time and material resources will be consumed by the calibrating experiments. Though data augmentation can be a supplement, the extra generation and validation operations still remain a burdensome undertaking. Therefore, several researchers have explored semi-supervised learning approaches to mitigate requirements for labeled data, and then alleviate experimental demands [123,147]. Additionally, the Physics-Informed Neural Network has demonstrated its potential in reducing required training data, which may become an emerging scheme for sensor calibration [148,149].
(2) Scaling high-precision calibration from single devices to large device populations. Though AI methods have effectively addressed many calibration tasks, current training processes typically focus on individual sensing systems. When confronted with large device populations, the approximate transfer learning methods remain the most common strategy, which is only practical for the LCSs without strict calibration demands. If the training outcomes could be fully leveraged in the following high-precision calibrations, the calibration costs of high-grade sensors would be greatly reduced, and the feasible applications would be further expanded. Current attempts in exploring features from historical databases may be a potential solution, but the establishment of such comprehensive historical databases still needs persistent resource investment.
(3) On-site, on-device calibration. Another challenge for AI models concerns the available computational resources, particularly for the calibration based on very complex models. The routine sensing platform can support the employment of many trained AI models in sensor calibration, but cannot provide adequate support for the model training, making the on-site, on-device calibration difficult to achieve. Transferring the model training from the ordinary consumer-grade computers to low-resource sensing nodes becomes another key for the wide applications of AI-based calibration. Researchers have attempted to optimize the data processing capability of miniaturized models to realize a trade-off between calibration accuracy and available resources [114,150,151]. Additionally, the teacher–student network architecture can transfer the knowledge of a complex network to a much simpler network, providing a viable solution for deploying high-precision models on limited computational resources in practical applications [152].
(4) Explainable models for calibration. Currently, the AI models often operate as a black-box system, and the entire training and learning process is opaque to researchers. First, this black-box data-driven approach precludes model-associated uncertainty quantification, thereby failing to fully satisfy the official definition of calibration. Then, this will prevent researchers from accessing or interpreting the knowledge acquired by AI models, and potentially constrain the accumulation of knowledge and experience in this domain. Meanwhile, the enclosed AI models also limit the human involvement in model optimization, which is a great stumbling block in the realization of “human + AI” intelligence. Fortunately, explainable AI has become a prominent research frontier with continuous breakthroughs [153]. It is anticipated that the opening AI models will also emerge in the sensor calibrations, aligning with the concurrent advancement of calibration technologies.

## 6. Conclusions

AI methods have played a significant role in sensor calibration. By leveraging the superior regression capabilities of AI models, researchers have successfully revealed the implicit mapping relationships in the input–output data pairs, providing an accurate and stable transfer function for the calibrated sensors. The ambient interferences are recognized and compensated for to maintain the sensing characteristics across various environments. Moreover, solutions for the calibration of large-scale, low-cost sensors are also realized by trained AI models, along with the feasible compensations of characteristic drift induced by prolonged usage. Several auxiliary tools, including data preprocessing, data augmentation and optimization of model training, have played remarkable roles in promoting the performance of AI models. Obviously, AI methods have accelerated the pace of innovation for sensor calibration. Despite these advances, current research still faces challenges in labeled data acquisition, rapid calibration of large-scale high-performance devices, on-site and on-device calibration, and the development of explainable AI models. Great efforts and resource investment are needed to render AI models a more powerful game-changer for sensor calibration.

## Figures and Tables

**Table 1:** Summery for the mentioned AI methods in sensor calibration.

Calibration Type		Target Sensor	Utilized AI Model	Data Number/Source	Gained Performance	Ref.
Transfer function	Single-to-single calibration	Pt RTD	BPNN	880	R^2^ = 0.9999873 for linearity, −50~150 °C	[46]
		K-type thermocouple	DFNN	1473	Nonlinearity from 2.03% to 0.002%, −100~1372 °C	[47]
		NTC thermistor	DLR-NN	121	Nonlinearity from 84.63% to 0.13%, 0~120 °C	[47]
		Capacitive pressure	SVR with RBF kernel	1000	R^2^ = 0.987 for linearity, 90–120 kPa	[48]
		Antenna for Temperature	LSTM	28 datasets	R^2^ = 0.997 for linearity, 20~170 °C	[39]
		PM2.5 sensor	k-Nearest Neighbors	5760	R^2^ = 0.964, RMSE = 2.194, MAE = 0.817	[58]
		CO_2_ sensor	Gradient Boosting	5760	R^2^ = 0.970, RMSE = 0.442, MAE = 0.282	[58]
		CO_2_ NDIR sensor	Stack ensemble ML	-	RMSE from 22.57 ppm to 9.53 ppm	[43]
		Vitamin C sensor	Two-step model based on CatBoost	-	LoD = 0.09 μM, R^2^ = 0.9962, and a linear range of 0.28~11.36 μM	[59]
		O_3_ sensor	Temporal pattern-based denoising and calibration with SVD	Public datasets for O_3_ MOX and O_3_ EQ sensors	Increase the calibration accuracy by 20–40%	[60]
	Coupled calibration	Flexible six-axis force/torque sensor	BPNN/DNN	2884	Class I error: from 2.9% F.S. to1.27% F.S. (BPNN) and 0.751% F.S. (DNN) Class II error: from 8.79%F.S. to 1.02% F.S (BPNN) and 0.603% F.S. (DNN)	[67]
		Multi-axis wheel force sensor	Improved decoupling algorithm with Informer network	-	Cross coupling error: from 16.18% F.S. to 1.32% F.S.	[68]
		Six-axis force/torque sensor	Multilayer perceptron	32,441	RMSE = 10.6 mN, MAE = 7.4 mN and a detectable force < 1 m N in the 284 mN range	[69]
		Hazardous odorimeter	Tailored TinyML	A total of 2500 measurements	Overall recognition accuracy of 0.9996	[72]
		12-sensor e-nose	LeNet-5	1000 grayscale response graph	Gas identification accuracy rate of 98.67%	[73]
Compensation	Intrinsic error	IMU	ML-ANFIS	-	54.23% and 51.14% reduction in RMSE azimuth compared to t XBOW RISS/GPS and radar RISS/GPS	[84]
		IMU	Tailored ALiMU	-	RMSE is improved by 49.845~99.080% for different IMUs	[85]
	Temperature	Laser methane sensor	BPNN + ISSA	15,810 at different temperatures	Relative error for CH_4_ concentration is 0.32% in −20~65 °C	[88]
		Silicon resonant accelerometer	BPNN + IFA	Output at four acceleration states of ±0 g, ±1 g in 30 s	Zero-bias stability × 10, scale factor stability × 70 in −40~60 °C	[89]
		Capacitive accelerometer	AGA-BPNN	-	Accuracy improved by 3.5% compared to GA-BP	[90]
		Fiber optic gyroscope	LSTM	-	Output was stabilized at 20°/s under a −3 °C/min impact	[95]
		Quartz resonant accelerometer	LSTM + Variable Coefficient Regression	-	Temperature coefficients of scale factor and zero bias are greatly improved from 18,104 ppm/°C to 0.773 ppm/°C and 6713.5833 μg/°C to 3.5833 μg/°C in −40~80 °C	[99]
		Micromechanical gyroscope	LSTM-SVM-DBN	Data from −40 ~100 °C with 20 °C intervals	Rate random wander and bias instability are reduced by 84.35% and 95.57% in −40~100 °C	[96]
		Methane emission sensor	ANN with three hidden layers	Output from 32 sensors	ppm errors are reduced to <1 ppm under +2 °C and +2.0% RH	[100]
	Degeneration drift	O_3_ sensor	Gradient Boosting	165- and 239-day test results	R^2^ is improved by 250%, estimation error is reduced by 90%	[108]
		E-nose device with 16 chemical sensors	Neural network with a CFE	3-year dataset with 13,910 measurements	RMSE is reduced by 90%	[109]
		16 screen-printed metal–oxide–semiconductor gas sensors	CNN	A public dataset with 13,910 measurements	Detection accuracy is maintained beyond 80% in three years	[111]
Large-scale LCS		Low-cost NO_2_, CO and O_3_ sensors	Random forest	60-month IRCELINE official data with 49,000 samples for training and a 2900-sample LCS dataset for testing	*R*^2^ = 0.72, RMSE = 0.0028 ppm	[115]

## Data Availability

Not applicable.
